# Tolerance of *Eugenia dysenterica* to Aluminum: Germination and Plant Growth

**DOI:** 10.3390/plants8090317

**Published:** 2019-08-31

**Authors:** Arthur Almeida Rodrigues, Sebastião Carvalho Vasconcelos Filho, Caroline Müller, Douglas Almeida Rodrigues, Juliana de Fátima Sales, Jacson Zuchi, Alan Carlos Costa, Cássia Lino Rodrigues, Adinan Alves da Silva, Danilo Pereira Barbosa

**Affiliations:** 1 Laboratory of Plant Anatomy, Goiano Federal Institute of Education, Science and Technology (IFGoiano), Campus Rio Verde, PO Box 66, Rio Verde, Goiás 75901-970, Brazil; 2 Laboratory of Ecophysiology and Plant Productivity, Goiano Federal Institute of Education, Science and Technology (IFGoiano), Campus Rio Verde, PO Box 66, Rio Verde, Goiás 75901-970, Brazil; 3 Laboratory of Seeds, Goiano Federal Institute of Education, Science and Technology (IFGoiano), Campus Rio Verde, PO Box 66, Rio Verde, Goiás 75901-970, Brazil

**Keywords:** accumulation, Al tolerance, cerrado, cagaita, root growth, Al pollution

## Abstract

Native Cerrado plants are exposed to soils with low pH and high availability of Al. In this study, we measured the Al content in adult plants, and investigated the effects of various Al doses on germination and early development of *Eugenia dysenterica* plants. For germination tests, the seeds were soaked in Al solution and evaluated for twenty days in growth chambers. In a second experiment, young plants were cultivated in hydroponic systems with various Al concentrations to evaluate the morphological, anatomical and physiological characteristics of *E. dysenterica*. Anatomical changes and low germinative vigor were observed in seeds germinated in 600 and 800 μmol Al^3+^ L^−1^. In the hydroponic system, 200 μmol Al^3+^ L^−1^ stimulated root growth in young plants. The activity of antioxidant enzymes and the accumulation of phenolic compounds were greatest at the highest Al doses, preventing changes in gas exchange and chlorophyll *a* fluorescence. Starch grain accumulation was noted in plant cells exposed to 200 and 400 μmol Al^3+^ L^−1^. Adult *E. dysenterica* trees also accumulated Al in leaves, bark and seeds. These data suggest that *E. dysenterica* is tolerant to Al.

## 1. Introduction

Aluminum (Al) toxicity is a limiting abiotic stress factor for many plants worldwide [1,2]. In soils with pH values below 5, Al becomes soluble by modifying its Al(OH)_3_ hydroxide form to toxic forms such as Al^3+^ [3,4]. In addition to being naturally abundant in acid soils, gradual increases in Al content in soils and waters have been reported, attributed to intense industrial metallurgy, packaging, transportation, construction, electrical, and chemical plant activities. These industries discard about 5 million tonnes aluminum-rich wastes per year worldwide [5,6,7]. Consequently, agricultural areas close to industries and use of industrial wastewater rich in aluminum are concerns for the cultivation of agricultural crops and the development of native species in these regions [7,8].

Among Al-sensitive species, some trees, including *Fraxinus excelsior* and *Acer pseudoplatanus*, are not able to complex Al via organic acids released by the root system [2]. In these species, Al inhibits root growth and secondary root formation [9] and damages mesophyll leaf cells [10], leading to inhibition of photosynthesis [11,12]. Some native plants from tropical regions with acidic and nutrient-poor soils have evolved survival strategies to deal with high Al saturation, in addition to acid and nutritional conditions; these species include some belonging to the Rubiaceae (*Melaleuca cajuputi* and *Coccocypselum sp.*) [13], Melastomataceae (*Melastoma malabathricum*) [14], and Vochysiaceae *(Qualea grandiflora*, *Callisthene major* and *Vochysia pyramidalis*) [15] families that can accumulate up to 10,000 mg Al Kg^−1^ in their leaves [14]. These species possess mechanisms for Al exclusion and/or internal complexation, permitting survival even at high Al concentrations [16,17]. Some Al-tolerant plants may accumulate between 1000 and 15,000 mg of Al^3+^ per kg of dry matter [18,19]. For these species, plant cultivation at concentrations between 320 and 530 μM Al^3+^ can stimulate root growth [20,21]. 

Industrial activity has gradually modified soil and water conditions in the vicinity of factories. There are a few studies on the relationship between Al and seed germination, as well as physiological and anatomical characteristics of native Brazilian Cerrado plants [22,23]. However, even plants considered to be Al-tolerant and Al-accumulators may suffer Al toxicity effects in conditions of continuous exposure to Al released by industrial processes [22]. 

*Eugenia dysenterica* DC is a native Brazilian Cerrado species from the Myrtaceae family, popularly known as cagaita [24,25]. The fruit of the plant has substantial economic potential [26]. Investigation of native Cerrado species with potential for high tolerance to Al is essential to understand these tolerance mechanisms. Such knowledge is also useful for the preservation of species under excessive Al conditions, in natural or even in contaminated environments [27,28]. Therefore, the study aimed to evaluate various Al concentrations (i) on seed germination and seed anatomical traits and, (ii) morphoanatomical and physiological traits in young plants of *Eugenia dysenterica* grown under a hydroponic system.

## 2. Material and Methods

### 2.1. Plant Material, Growth Conditions and Al Treatments 

*E. dysenterica* seeds were obtained from the fruits of 15 adult plants in full production in an uncultivated area of the Cerrado, located in the rural area of the municipality of Montes Claros, Goiás, Brazil (latitude 16° 06′20″ S - longitude 51° 17′ 11″ W, altitude of 592 m). A specimen was deposited at the Goiano Federal Institute Herbarium, Rio Verde Campus under number 630/2017.

### 2.2. Germination Test

The seeds of the pulped fruit were separated and arranged in a linear and alternate manner on two Germitest paper sheets moistened with a solution containing five Al concentrations (0 (control), 200, 400, 600 and 800 μmol L^−1^ of Al_2_(SO_4_)_3_·18H_2_O) in a calcium chloride solution (CaCl_2_ 0.1 mM, pH 4.0). Calcium chloride solution only was used as the control. The Germitest paper was moistened with 2.5 times its dry weight, and the rolls were then packed in transparent plastic bags and maintained in a Mangelsdorf-type germinator at the constant temperature of 25 °C (±0.5 °C) and photoperiod of 12 h. 

The seeds were recorded as germinated when root protrusion achieved 2 mm. Readings were performed daily to calculate the germination percentages and germination rate index (GRI) [29], according to the formula: GRI = G1/N1 + G2/N2 + … + Gn/Nn; where G is the number of normal seedlings observed each day and, N is the number of days.

Root diameter measurements on germination were performed at 35 days after sowing (DAS) at a height of one centimeter at the base of the stem. The experiment comprised five treatments (Al^3+^ concentrations) and four replicates, each replicate consisting of 25 *E. dysenterica* seeds.

### 2.3. Morphoanatomical Seed Characterization 

*E. dysenterica* seeds were treated as described in Section 2.2, in a completely randomized design. On the 20th day of treatment, 3 cm^2^ samples from the endosperm region of three seeds per replicate (n = 4) were collected per treatment (n = 5). The samples were first fixed in Karnovsky solution [30] for 24 h. Subsequently, they were prewashed in a phosphate buffer (0.1 M, pH 7.2) and dehydrated in an increasing ethanol series (30% to 100%), pre-infiltrated and historesin infiltrated (Leica, Germany) according to manufacturer’s recommendations. Subsequently, the samples were cross-sectioned at 5 μm thickness using a rotary microtome (Model 1508R, Logen Scientific, China) and stained with toluidine blue polychromatic coloration (0.05% 0.1 M phosphate buffer, pH 6.8) [31]. Starch detection was performed using histochemical staining with Lugol solution at 10 g L^−1^ [32]. Images were obtained under an Olympus microscope (BX61, Tokyo, Japan), coupled to a DP-72 camera, using the clear field option.

### 2.4. Hydroponic Young Plant Growth

Initially *E. dysenterica* seeds were sown in beds containing washed sand as the substrate. Approximately 40 days after emergence, two seedlings of standard height (~14 cm) were transplanted per plastic vat containing 1.5 L of a calcium chloride solution (CaCl_2_ 0.1 mM, pH 4.0) at low ionic strength. The solution pH was adjusted to 4.0 using 1 M HCl and 0.1 M NaOH solutions. After 10 days of acclimation in a greenhouse, the plants were exposed to five Al concentrations (0 (control), 200, 400, 600 and 800 μmol L^−1^), in the form of aluminum sulphate (Al_2_(SO_4_)_3_·18H_2_O) as described by Tolrà et al. [33] and Rodrigues et al. [34]. The solution was maintained under aeration of 100 cm^3^ min^−1^ air pressure and was renewed every three days.

The experiment was performed for 20 days, in a completely randomized design, with controlled conditions, monitored by an SKDL-32 data logger containing a temperature and relative humidity sensor, at a mean relative humidity of 65% (±5) and mean temperatures of 27 °C (±5, day) and 22 °C (±5, night) in a greenhouse at the Laboratory of Ecophysiology and Plant Productivity. 

#### 2.4.1. Visible Root and Leaf Symptoms 

Visible symptoms were recorded photographically. Fully expanded leaf surfaces and the root system of the plants at the end of the experimental period were photographed with a digital camera (Cyber-Shot SONY HX100V, Japan). Images covered the leaf and root that best represented each treatment.

#### 2.4.2. Root Growth Measurements

Root measurements were performed daily during the 20 days of Al plant exposure. At the end of the exposure period, measurements of the main root were taken and the total root growth rate (TRG) was calculated as (TRG = [(Cf/Ci)*100]-100, where Ci and Cf indicate the initial and final root lengths, respectively). Relative root elongation (RRE%) was calculated according to the equation proposed by Vasconcelos et al. [35]: (RRE = [(CfAl_x_ − CiAl_x_)*100]/(Cf Al_0_ − CiAl_0_). Where CiAlx: initial root length measured before exposure to the nutrient solution at an “x” level of Al; CfAlx: final root length measured after n days of exposure to the nutrient solution at an “x” level of Al; CiAl_0_: initial root length before exposure to the solution without any Al; and CfAl_0_: final root length measured after n days of exposure to the nutrient solution without Al.

#### 2.4.3. Chlorophyll a Fluorescence 

Chlorophyll *a* fluorescence variables were determined in the last fully expanded leaf using a fluorometer (6400-40, Li-color, Nebraska, USA) coupled to an IRGA (IRGA, LI-6400xt, Li-Cor, Nebraska, USA). Initially, the leaves were adapted to the dark for at least 30 min (when the photosystem II (PSII) reaction centers were open), followed by the application of the measurement light (~0.03 µmol m^−2^ s^−1^) and a saturation pulse (>3000 µmol m^−2^ s^−1^), used to obtain the minimum (F_0_) and maximum (F_m_) fluorescence, respectively. The potential quantum yield of the PSII was determined as F_v_/F_m_ = (F_m_-F_0_)/F_m_ [36]. After lighting with a continuous actinic light source (~1000 µmol m^−2^ s^−1^) for 40 s, a saturation pulse was applied to determine the maximum fluorescence (F_m_′) and steady state (F_s_) in light-adapted leaves. The data were used to calculate the effective quantum yield of the PSII (ΔF/F_m_′= [F_m_′-F_s_]/F_m_′), the photochemical extinction coefficient (qP = [F_m_′-F]/[F_m_′-F_o_′]), the non-photochemical extinction coefficient (NPQ = [F_m_-F_m_′]/F_m_′), the apparent rate of electron transport (ETR = Φ PSII x RFA x 0.5 × 0.84), and the maximum photochemical PSII efficiency in light-adapted leaves (F_v_′/F_m_′ = [(F_m_′–F_o_′)/F_m_′]) [37].

#### 2.4.4. Gas Exchange

Gas exchange was assessed on the same leaf as the chlorophyll *a* fluorescence data to determine photosynthetic rate (*A*, μmol m^−2^ s^−1^), stomatal conductance (*g*s, mol H_2_O m^−2^ s^−1^), transpiration rate (*E*, mmol m^−2^ s^−1^), ratio of internal to external CO_2_ (*Ci*/*Ca*), and the ratio of photosynthetic rate to internal CO_2_ concentration (*A*/*Ci*). Measurements were performed using an infrared gas analyzer (IRGA, LI-6400xt, Li-cor, Nebraska, EUA). Assessments were performed between 9:00 AM and 11:00 AM under constant photosynthetically-active radiation (PAR, 1000 μmol of photons m^−2^ s^−1^) and CO_2_ concentration (~415 μmol mol^−1^), ambient temperature (~25.5 °C), and relative humidity (~74%).

#### 2.4.5. Morphoanatomical Root and Leaf Characterization 

For the morphoanatomical analyses, 3–5-cm root and 3 cm^2^ leaflet *E. dysenterica* samples were collected from the root tip and from the last fully expanded leaf of all replicates (n = 4) from each treatment (n = 5) after 20 days of hydroponic cultivation in Al-containing solutions. The material was washed and processed as described in Item 2.3. The plant material was stained with toluidine blue to obtain epidermis images for morphoanatomical observations, i.e., the adaxial and abaxial surfaces, palisade and spongy parenchyma, other mesophyll tissues and the meristematic root region. Starch staining was performed using histochemistry with Lugol solution at 10 g L^−1^ [32].

### 2.5. Al Content Quantification

Al content was determined in both adult trees from which fruits and seeds were collected and from experimental plants. Leaf and bark samples were collected from five adult plants in full production. Al content was also evaluated in *E. dysenterica* seeds after twenty days of exposure to various Al concentrations and in young leaves and roots after twenty days of growth in a nutrient solution containing various Al concentrations.

The collected material was previously washed in distilled water to remove adhered Al, dried in a circulation oven, heated at 70 °C for 78 h, and ground in a Wiley mill (3383-L10, Thomas Scientific, USA). Plant samples (500 mg) were added to tubes containing a nitroperchloric solution (2:1) and were digested in a digester block at 160 °C. Subsequently, the tube volume was brought to 25 mL with deionized water, as described by Malavolta et al. [38] and Al contents were determined on a plasma-coupled optical emission spectrometer (OPTMA 7300 DV, Perkin Elmer). Aluminum concentrations were expressed as mg kg^−1^.

### 2.6. Hydroponic culture: antioxidant enzyme activity

Superoxide dismutase (SOD, EC 1.15.1.1), catalase (CAT, EC 1.11.1.6) and peroxidase (POX, EC1.11.1.7) activities were determined by preparing plant extracts using maceration of approximately 300 mg of roots in 2 mL of an extraction medium, consisting of 0.1 M potassium phosphate buffer, pH 6.8; 0.1 mM ethylenediaminetetraacetic acid (EDTA); 1 mM phenylmethylsulfonic fluoride (PMSF) and 1% (w/v) polyvinylpolypyrrolidone (PVPP). The maceration solution was subjected to centrifugation at 12,000 x *g* for 15 min at 4 °C and the supernatant (enzyme extract) was used for the enzymatic determinations.

SOD activity was determined by adding 40 μL of the enzyme extract to 5 mL of the reaction medium, consisting of a 50 mM sodium phosphate buffer, pH 7.8, containing 13 mM methionine, p-nitro tetrazolium blue (NBT) 75 μM, 0.1 mM EDTA and 2 μM riboflavin. The reaction was conducted at 25 °C in a chamber under illumination of a 15 W fluorescent lamp for 10 min. Blue formazan absorbance produced by the NBT photoreduction was determined at 560 nm [39]. The results were expressed as units of SOD (U SOD) min^−1^ mg^−1^ protein. One unit of SOD was defined as the amount of enzyme required to inhibit NBT photoreduction by 50% [40].

Catalase activity was determined by adding 0.1 mL of the enzyme extract to 2.9 mL of the reaction medium, consisting of a 50 mM potassium phosphate buffer pH 7.0 and 12.5 mM H_2_O_2_ [41]. The decrease in absorbance by H_2_O_2_ degradation was determined at 240 nm in the first minute of the reaction at 25 °C. Enzymatic activity was calculated using the molar extinction coefficient of 36 M^−1^ cm^−1^ [42] and was expressed as μmol min^−1^ mg^−1^ protein.

Peroxidase activity was determined by the addition of 0.1 mL of the enzyme extract to 4.9 mL of the reaction medium, consisting of a 25 mM potassium phosphate buffer pH 6.8, 20 mM pyrogallol and 20 mM H_2_O_2_ [43]. Purpurogallin production was determined by the increasing absorbances during the first minute of the reaction at 420 nm at 25 °C. Enzymatic activity was calculated using a molar extinction coefficient of 2.47 mM^−1^ cm^−1^ [44] and was expressed as μmol min^−1^ mg^−1^ protein.

The protein content in the enzymatic extracts was quantified according to the methodology proposed by Bradford [45] at 595 nm. The results were compared to a standard bovine serum albumin (BSA) curve and used to express enzymatic activity on a protein basis.

### 2.7. Statistical Analyses

The quantitative data were first subjected to homogeneity analysis (Levene test) and error normality assessment (Shapiro-Wilk test). Because data normality was confirmed, ANOVA was performed, followed by Dunnett’s test, to determine significant differences between the Al treatments and the control (*p* < 0.05). All statistical analyses were performed using ASSISTAT v. 7.7 software.

## 3. Results

### 3.1. Germination

The germination rate index (GRI) and germination percentages in *E. dysenterica* decreased with increasing Al^3+^ concentrations (Table 1). The highest Al^3+^ dose led to a 70.25% decrease in the GRI and 30% decrease in germination percentage compared to the control (Table 1).

### 3.2. Germination: Anatomical Seed Changes 

**Figure 1:**
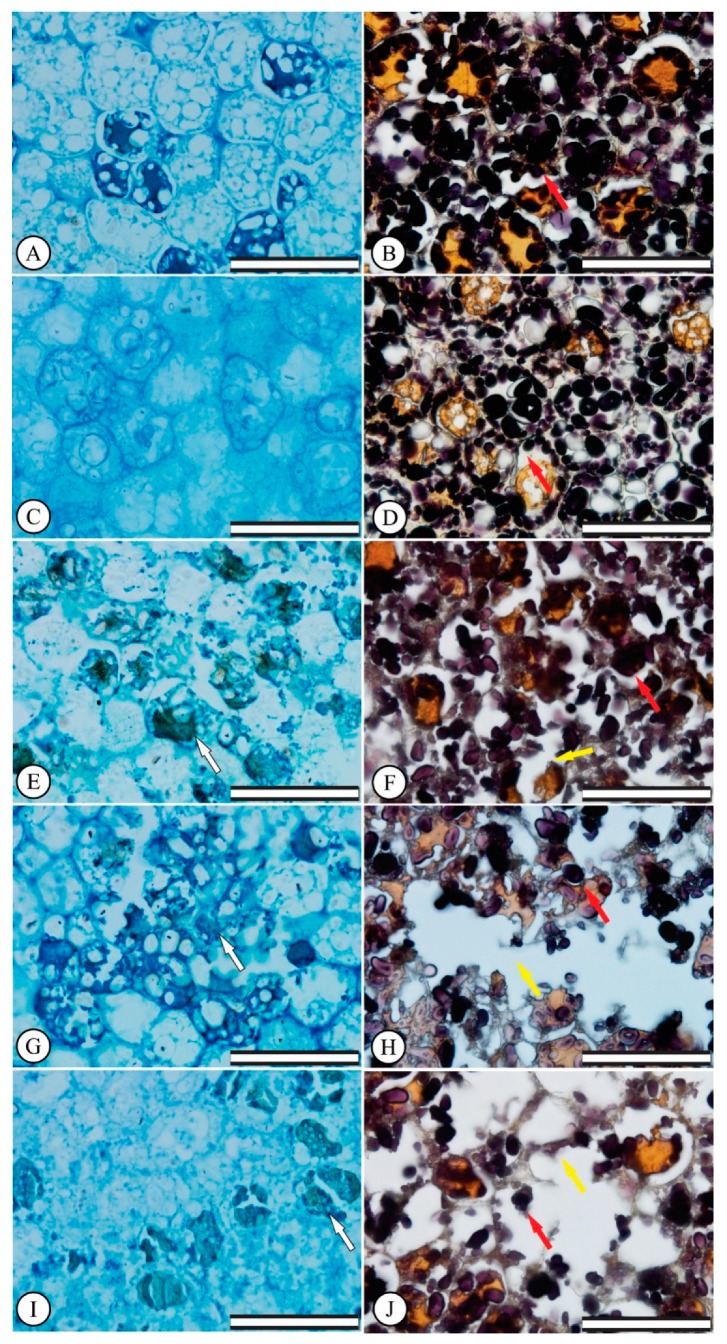
*Eugenia dysenterica* seed endosperms after the germination test. (**A**,**B**) control treatment, (**C**,**D**) 200 μmol L^−1^ Al^3+^, (**E**,**F**) 400 μmol L^−1^ Al^3+^, (**G**,**H**) 600 μmol L^−1^ Al^3+^, (**I**–**J**) 800 μmol L^−1^ Al^3+^. White arrows indicate phenolic compound production, red arrows indicate starch accumulation and yellow arrows indicate cell disruption. (First column) Scale bar 50 μm. (Second column) Scale bar 100 μm.

Increasing Al^3+^ doses caused endosperm region cell destruction in *E. dysenterica* (Figure 1C,G,E–I) when compared to the control (Figure 1A). The green coloration revealed by toluidine blue indicated the presence of phenolic content in some endosperm region cells at 400, 600 and 800 μmol L^−1^ Al^3+^ (Figure 1E,G–I). Regarding starch accumulation, the control cells presented large areas marked by Lugol stain in the endosperm region of the seeds (Figure 1B), while increasing Al^3+^ doses at 400, 600 and 800 μmol L^−1^ led to starch grain extrusion through the cells, via endosperm cell disruption and collapse.

### 3.3. Hydroponic Culture: Visible Morphological Symptoms

**Figure 2:**
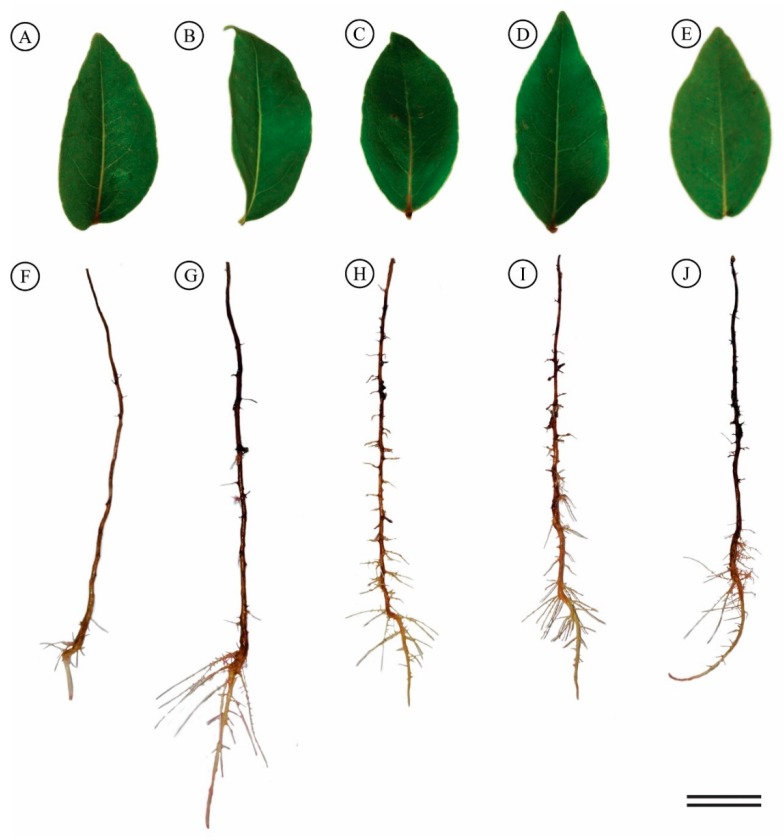
Visual appearance of *Eugenia dysenterica* leaves and roots after 20 days exposure to various Al concentrations: (**A**–**F**) control, (**B**–**G**) 200, (**C**–**H**) 400, (**D**–**I**) 600 and (**E**–**J**) 800 μmol L^−1^ AL. Bar = 2 cm.

Visual leaf assessments in response to the various Al treatments did not show toxicity or nutritional deficiency symptoms (Figure 2A–E). Greater root growth was observed with 200 μmol L^−1^ Al^3+^ than control and other Al treatments (Figure 2F–G). Although no growth changes were observed in the other treatments, stimulation of secondary root formation was noted (Figure 2).

The seedlings subjected to 200 μmol L^−1^ of Al^3+^ displayed increased mean root length (36%), root growth rate (58%) and relative root length (30%) than the control seedlings (Table 2), also observed for the 600 and 800 μmol L^−1^ treatments (Table 2).

### 3.4. Hydroponic Culture: Anatomical Seedling Changes 

**Figure 3:**
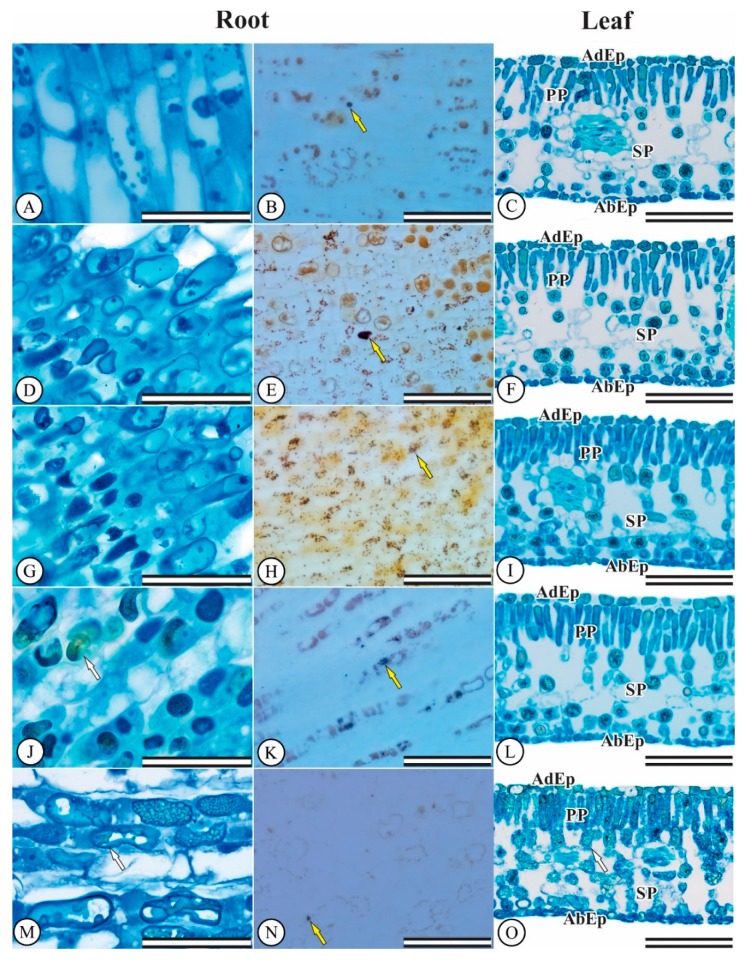
*Eugenia dysenterica* root and leaf anatomy after 20 days of growth at various Al concentrations. (**A**–**C**) control treatment, (**D**–**F**) 200 μmol L^−1^ Al^3+^, (**G**–**I**) 400 μmol L^−1^ Al^3+^, (**J**–**L**) 600 μmol L^−1^ Al^3+^, (**M**,**N**,**O**) 800 μmol L^−1^ Al^3+^. (AdEp) adaxial epidermis. (AbEp) abaxial epidermis. (PP) palisade parenchyma. (SP) spongy parenchyma. (**A**,**B**,**D**,**E**,**G**,**H**,**J**,**K**,**M**,**N**) root meristem and differentiation zone. (**C**,**F**,**I**,**L**,**O**) leaf mesophyll cells. White arrows indicate the production of phenolic compounds and yellow arrows indicate starch accumulation. (First column) Scale bar = 50 μm. (Second and third columns) Scale bar = 100 μm.

The *E. dysenterica* epidermis is unstratified on both surfaces. The stomata are present only on the abaxial surface, characterizing leaves as hypostomatic. In addition, they are located at the same level as other epidermal cells. The chlorophyllic parenchyma is typically dorsiventral, the palisadic parenchyma consists of only one cell layer and the spongy parenchyma consists of about six layers. The greenish coloration revealed by toluidine blue indicates the presence of phenolic content in some cells in the filling parenchyma region, mainly for the 600 and 800 μmol L^−1^ Al^3+^ treatments. Al did not affect root meristem and differentiation zone (Figure 3A,D,G), and leaf mesophyll cells (Figure 3C,F).

Regarding root system starch accumulation, control cells displayed small Lugol-stained areas (Figure 3B). Starch grain accumulation was noted in plant cells exposed to 200 and 400 μmol L^−1^ Al^3+^ (Figure 3E–H). The 600 and 800 μmol L^−1^ treatments led to lower levels starch cell accumulation (Figure 3K–N).

### 3.5. Hydroponic Culture: Chlorophyll a Fluorescence and Gas Exchanges

Regarding *E. dysenterica* chlorophyll *a* fluorescence parameters, only the effective quantum yield of the PSII (ΔF/F_m_′) was altered, with a 16.07% increase observed for the 200 μmol L^−1^ Al^3+^ treatment in relation to the control (Table 3).

Net photosynthetic rate (*A*), stomatal conductance (gs), transpiration rate (*E*), internal to external CO_2_ concentration ratio (*Ci*/*Ca*) and photosynthetic rate to internal CO_2_ concentration (Ci) ratio (*A*/*Ci*) were not affected by Al in *E. dysenterica* plants (Table 3).

### 3.6. Hydroponic Culture: Antioxidant Enzyme Activity

Antioxidant enzyme activities were differentially modulated by Al in *E. dysenterica* seedling roots. Increasing Al concentrations increased CAT and POX activity, mainly at 200 and 400 μmol L^−1^ Al^3+^ (Table 4). SOD activity was increased by 36.66% compared to the control at 800 μmol L^−1^ Al^3+^ (Table 4).

### 3.7. Al Content 

Adult *E. dysenterica* trees were found to be Al accumulators, accumulating Al in bark (≥1000 mg kg^−1^), and also able to accumulate high Al content in leaves (≥552.64 mg kg^−1^). Seeds inherited Al in their tissues (111.07 mg kg^−1^) from their parent plants and increasing Al doses in the germination test led to higher Al content found in seeds, although this was significantly different only for 800 μmol L^−1^ Al^3+^ (Table 5). In the hydroponic cultivation experiment, *E. dysenterica* plants were shown to contain Al in roots (521.04 mg kg^−1^) and leaves (140.94 mg kg^−1^), even in the absence of Al in the growth solution. Roots abundantly accumulated Al in all treatments, reaching 2332.46 mg kg^−1^ in the 800 μmol L^−1^ Al treatment. Similar results were verified for leaves; however, the amount of accumulated Al was much lower compared to the values in roots.

## 4. Discussion

The phytotoxic action of Al on *E. dysenterica* seeds altered the germination process, compromising embryo development through endosperm cell destruction and solute extrusion. Despite the fact that *E. dysenterica* matrix plants store Al in seeds, external contact with high Al concentrations may interfere with germination and may impair root growth and seedling establishment. The hypothesis we proposed was that seeds would not present Al cell detoxification mechanisms or complexation processes against absorption, greatly increasing the direct interference of Al on cell division, inhibiting germination. In this manner, direct environmental Al contact with *E. dysenterica* seeds would lead to impaired germination processes.

The low seed germination levels in the presence of Al suggests that the amount of Al accumulated in seeds is sufficient to inhibit embryo growth resumption and seedling formation [22]. In this sense, exogenous application of Al may have led to a certain degree of embryonic toxicity to *E. dysenterica*, affecting cell division and/or elongation and root protrusion. Toxic effects of Al have also been observed in *Conyza* seeds, with a 35% decrease in *Conyza canadensis* and 60% in *Conyza bonariensis* seed germination [46]. Moreover, similar to our results, Koszo et al. [47] found that Al compromised processes that preceded the primary root protrusion in the *Erythrina speciosa* and *Eugenia brasiliensis* seeds. This suggests that Al compromises seed vigor, even in tolerant species, resulting in seedlings with less robust root systems, as observed in the present study.

*E. dysenterica* plants grown at various Al concentrations did not display morphological, anatomical or physiological damage; rather, root growth was stimulated by the 200 μmol L^−1^ Al treatment. This root growth increment under low Al concentrations was previously reported for *E. dysenterica* by Rodrigues et al. [21], who postulated that this was an adaptation to Al-rich acid soils. When toxic to plants, Al is associated with abrupt root morphology changes characterized by the production of smaller, thick apices with a darker coloration and low formation of secondary roots [48]. This suggests that Al concentrations were not toxic to *E. dysenterica* roots in the hydroponic assay. Corroborating the findings of the present study, Rodrigues et al. [34], when assessing the tolerance potential of *Hancornia speciosa* grown in a nutrient solution, observed root growth stimulation at 300 μmol L^−1^ Al. These responses may be associated with Al detoxification via phenolic compounds and starch accumulation, increased root nutrient uptake and the formation of Al detoxification mechanisms.

The greater starch accumulation in *E. dysenterica* root system cells is a result of the inhibition of carbohydrate translocation, an energy source for plants that stimulates greater root growth. Påhlsson [49] reported starch content increases in *Fagus sylvatica* roots during 31 days of growth under Al treatment, with no root growth decreases. These data suggest that carbohydrate accumulation is associated with root growth stimulation caused by Al, attributable to the greater availability of energy sources such as starch and sucrose.

*E. dysenterica* mesophyll cell and root system integrity suggests that Al does not affect anatomical characteristics, because Al stress in sensitive plants usually leads to changes in leaf and root structures [10,50,51], while on the other hand, cellular structure preservation is associated with Al stress tolerance. Rodrigues et al. [21] reported that *E. dysenterica* absorbs Al by the root system; therefore, cell walls and vacuoles are the primary storage sites for accumulation, suggesting that the tolerance system is based on internal chelation and Al compartmentalization, instead of absorption restriction [33]. Reinforcing this theory, *E. dysenterica* phenol accumulation suggests an Al detoxification strategy and subsequent complexation with these metabolites [52]. Phenolic compounds act as antioxidants in plants under stress [53], and play a potential role in the exclusion of Al^3+^ [28].

In sensitive species, Al affects plant physiological characteristics such as net photosynthetic rate and stomatal conductance [11,54]. In the present study, *E. dysenterica* did not present changes to chlorophyll *a* fluorescence or gas exchange. Al-tolerant species may exhibit stimulated growth over a wide range of Al concentrations [12] with high photosynthetic activity and PSII photochemical efficiency and electron transport rate maintenance [55]. In addition, NPQ, a thermal dissipation indicator under stressful conditions [56] was not altered during Al cultivation, suggesting that the plants were not under stress.

The increase of the antioxidant capacity of *E. dysenterica* plants is related to the activation of a defense mechanism to protect plants against the formation of reactive oxygen species by the action of SOD, CAT and POX enzymes [57]. Increases in CAT and POX were observed in *E. dysenterica* cultivated with Al, mainly at 200 and 400 μmol L^−1^ Al^3+^, when root growth was stimulated. Similar results were reported by Ghanati et al. [58] in *Camellia sinensis* plants, where SOD and CAT activities in roots increased in the presence of Al, suggesting that these antioxidant enzymes are beneficial for tea plants and for stimulating root growth. SOD activity is associated with increased superoxide radical production, which is metabolized into hydrogen peroxide (H_2_O_2_) [59]. Several enzymes regulate intracellular H_2_O_2_ levels in plants; however, CAT and POX have higher affinities for H_2_O_2_ removal [60].

This species naturally accumulates Al in its tissues (roots, bark, leaves and seeds) when it is grown in soils containing Al. Metal accumulation, predominantly heavy metals, is a characteristic present in over 450 species of vascular plant families, orders and genera [61]. Native communities in savannas and tropical forests are rich in species that have evolved survival strategies to cope with restrictive edaphoclimatic conditions such as high soil acidity, high Al saturation and low nutrient availability [15]. Therefore, our results confirm that *E. dysenterica* can be characterized as an Al-tolerant species as demonstrated by root growth stimulation, anatomical integrity and maintenance of gas exchange and chlorophyll fluorescence parameters. Furthermore, Al promotes plant growth, primarily at lower concentrations.

## 5. Conclusions

*E. dysenterica* germination was affected by exposure to various Al concentrations. Nevertheless, young *E. dysenterica* plants showed Al tolerance. Anatomical and physiological traits were not changed by increasing Al doses. The accumulation of phenolic compounds and the activation of antioxidant enzymes system acted as Al-detoxification mechanisms in cells. Starch accumulation may be related with the highest root growth observed at 200 μmol L^−1^ Al^3+^.

## Figures and Tables

**Table 1:** Germination rate index (GRI) and germination percentage (%) in *Eugenia dysenterica* seeds after the application of a liquid calcium chloride solution containing different Al doses (0, 200, 400, 600 and 800 μmol L^−1^).

Al^3+^ Concentration (Al_2_(SO_4_)_3_	GRI	Germination (%)
0 μmol L^−1^	1.21 ± 0.09	80 ± 3.65
200 μmol L^−1^	1.14 ± 0.06	75 ^**^ ± 2.52
400 μmol L^−1^	1.08 ± 0.03	64 ^**^ ± 1.63
600 μmol L^−1^	0.87 ^**^ ± 0.05	61 ^**^ ± 3.79
800 μmol L^−1^	0.85 ^**^ ± 0.09	56 ^**^ ± 2.83
**F**	**	**
**CV%**	**6.39**	**4.29**

Means ± SE (n = 4), Asterisks indicate significant differences at 5% (*) and 1% (**) probability, relative to the control as indicated by Dunnett’s test.

**Table 2:** Mean root length (RL), total root growth rate (TRG%) and relative root elongation (RRE%) of the *Eugenia dysenterica* root system after 20 days grown in solution at various Al concentrations (0, 200, 400, 600 and 800 μmol L^−1^).

Al^3+^ Concentration (Al_2_(SO_4_)_3_	RL	TRG%	RRE%
0 μmol L^−1^	4.05 ± 0.24	18.92 ± 0.28	100.00 ± 0.00
200 μmol L^−1^	5.51 ^*^ ± 0.35	29.87 ^**^ ± 1.15	130.22 ^**^ ± 5.09
400 μmol L^−1^	4.17 ± 0.37	19.52 ± 0.69	103.57 ± 4.17
600 μmol L^−1^	4.23 ± 0.31	24.30 ^**^ ± 0.94	109.73 ± 4.23
800 μmol L^−1^	4.74 ± 0.36	25.42 ^**^ ± 1.51	85.06 ± 4.74
**F**	*	**	**
**CV%**	**16.21**	**9.51**	**11.53**

Means ± SE (n = 5), Asterisks indicate significant differences at 5% (*) and 1% (**) probability, relative to the control as indicated by Dunnett’s test.

**Table 3:** Photosystem II (PSII) quantum potential yield (F_v_/F_m_), effective quantum yield of the PSII when reaction centers are reduced (ΔF/F_m_′), and non-photochemical dissipation (NPQ). Net photosynthetic rate (*A*), stomatal conductance (*g*s), transpiration rate (*E*) and relation between the internal and external CO_2_ concentrations (*Ci*/*Ca*) in *Eugenia dysenterica* plants, after 20 days of growth at different Al concentrations (0, 200, 400, 600 and 800 μmol L^−1^). Electron transport (ETR).

	**Chlorophyll *a* Fluorescence Traits**			
**Al^3+^ Concentration (Al_2_(SO_4_)_3_**	**F_v_/F_m_**	**ΔF/F_m_′**	**ETR**	**NPQ**
0	0.74 ± 0.024	0.56 ± 0.01	245.24 ± 8.02	0.72 ± 0.05
200	0.68 ± 0.040	0.65 ^**^ ± 0.02	275.43 ± 13.37	0.52 ± 0.18
400	0.62 ± 0.060	0.60 ± 0.3	272.82 ± 20.58	0.65 ± 0.28
600	0.71 ± 0.040	0.63 ± 0.00	282.73 ± 9.95	0.64 ± 0.23
800	0.71 ± 0.027	0.51 ± 0.01	245.10 ± 17.21	1.01 ± 0.27
**F**	**NS**	**	**NS**	**NS**
**CV (%)**	**13.15**	**8.24**	**12.33**	**68.82**
	**Gas Exchange Traits**			
**Al^3+^ Concentration (Al_2_(SO_4_)_3_**	***A***	***gs***	***E***	***Ci*/*Ca***
0	9.12 ± 0.62	0.14 ± 0.02	1.39 ± 0.21	0.73 ± 0.021
200	7.55 ± 0.99	0.18 ± 0.03	1.72 ± 0.24	0.78 ± 0.020
400	7.23 ± 1.00	0.14 ± 0.03	1.37 ± 0.24	0.73 ± 0.053
600	7.98 ± 0.58	0.16 ± 0.04	1.49 ± 0.34	0.70 ± 0.015
800	7.67 ± 0.98	0.15 ± 0.04	1.36 ± 0.28	0.70 ± 0.039
**F**	**NS**	**NS**	**NS**	**NS**
**CV (%)**	**24.20**	**47.56**	**40.27**	**10.12**

Means ± SE (n = 5), Asterisks indicate significant differences at 5% (*) and 1% (**) probability, relative to the control as indicated by Dunnett’s test. (NS) non-significant.

**Table 4:** Antioxidant *Eugenia dysenterica* root system superoxide dismutase (SOD), catalase (CAT), peroxidase (POX) enzyme activities after 20 days of growth in a hydroponic culture at different Al concentrations (0, 200, 400, 600 and 800 μmol L^−1^).

Al^3+^ Concentration (Al_2_(SO_4_)_3_	SOD	CAT	POX
0 μmol L^−1^	43.02 ± 1.85	25.58 ± 2.55	0.52 ± 0.25
200 μmol L^−1^	58.79 ± 3.28	74.74 ^**^ ± 3.61	3.03 ^**^ ± 0.27
400 μmol L^−1^	40.31 ± 3.27	57.48 ^**^ ± 9.80	3.13 ^**^ ± 0.29
600 μmol L^−1^	30.37 ± 7.09	15.97 ± 0.85	2.33 ^**^ ± 0.32
800 μmol L^−1^	62.50 ^*^ ± 6.53	30.32 ± 4.41	2.26 ^**^ ± 0.09
**F**	*	**	**
**CV%**	**30.39**	**59.12**	**46.30**

Means ± SE (n = 5), Asterisks indicate significant differences at 5% (*) and 1% (**) probability, relative to the control as indicated by Dunnett’s test.

**Table 5:** *Eugenia dysenterica* Al content in seedlings (Al-Bark and root), seeds from the germination test (Al-Seeds), hydroponic roots grown in solution (Al-Root), leaves grown in solution (Al-Leaf) after 20 days of hydroponic growth at different Al concentrations (0, 200, 400, 600 and 800 μmol L^−1^).

	**Al-Bark from Adult Trees (mg kg^−1^)**	**Al-Leaves from Adult Trees (mg kg^−1^)**	
Plant 1	1188.25	588.05	
Plant 2	1027.25	543.80	
Plant 3	1080.95	549.02	
Plant 4	1433.32	515.23	
Plant 5	1356.87	567.21	
**Al^3+^ Concentration (Al_2_(SO_4_)_3_**	**Al-Seeds Germination Test**	**Al-root Hydroponic Cultivation**	**Al-Leaves Hydroponic Cultivation**
0 μmol L^−1^	111.07 ± 6.81	521.04 ± 13.66	140.94 ± 1.96
200 μmol L^−1^	123.33 ± 4.85	1984.23 ^**^ ± 15.78	195.14 ^**^ ± 2.84
400 μmol L^−1^	130.08 ± 14.13	2146.13 ^**^ ± 60.40	210.10 ^**^ ± 5.21
600 μmol L^−1^	132.60 ± 9.58	2300.67 ^**^ ± 54.13	228.39 ^**^ ± 10.20
800 μmol L^−1^	180.82 ^**^ ± 12.95	2332.46 ^**^ ± 37.70	239.62 ^**^ ± 2.47
**F**	**	**	**
**CV%**	**21.31**	**37.91**	**17.54**

Means ± SE (n = 5), Asterisks indicate significant differences at 5% (*) and 1% (**) probability, relative to the control as indicated by Dunnett’s test.
